# Design Strategies and Emerging Applications of Conductive Hydrogels in Wearable Sensing

**DOI:** 10.3390/gels11040258

**Published:** 2025-04-01

**Authors:** Yingchun Li, Shaozhe Tan, Xuesi Zhang, Zhenyu Li, Jun Cai, Yannan Liu

**Affiliations:** 1 Advanced Interdisciplinary Research Center for Flexible Electronics, Academy of Advanced Interdisciplinary Research, Xidian University, Xi’an 710071, China; 2 State Key Laboratory of Wide-Bandgap Semiconductor Devices and Integrated Technology, Faculty of Integrated Circuit, Xidian University, Xi’an 710071, China; 3 Shaanxi Key Laboratory of Degradable Biomedical Materials, School of Chemical Engineering, Northwest University, Xi’an 710069, China

**Keywords:** hydrogel, sensor, conductive, wearable, flexible electronic

## Abstract

Conductive hydrogels, integrating high conductivity, mechanical flexibility, and biocompatibility, have emerged as crucial materials driving the evolution of next-generation wearable sensors. Their unique ability to establish seamless interfaces with biological tissues enables real-time acquisition of physiological signals, external stimuli, and even therapeutic feedback, paving the way for intelligent health monitoring and personalized medical interventions. To fully harness their potential, significant efforts have been dedicated to tailoring the conductive networks, mechanical properties, and environmental stability of these hydrogels through rational design and systematic optimization. This review comprehensively summarizes the design strategies of conductive hydrogels, categorized into metal-based, carbon-based, conductive polymer-based, ionic, and hybrid conductive systems. For each type, the review highlights structural design principles, strategies for conductivity enhancement, and approaches to simultaneously enhance mechanical robustness and long-term stability under complex environments. Furthermore, the emerging applications of conductive hydrogels in wearable sensing systems are thoroughly discussed, covering physiological signal monitoring, mechano-responsive sensing platforms, and emerging closed-loop diagnostic–therapeutic systems. Finally, this review identifies key challenges and offers future perspectives to guide the development of multifunctional, intelligent, and scalable conductive hydrogel sensors, accelerating their translation into advanced flexible electronics and smart healthcare technologies.

## 1. Introduction

Flexible electronic sensors, serving as pivotal components of next-generation intelligent perception systems, have demonstrated transformative potential in wearable health monitoring, human–machine interfaces, and soft robotics [1,2,3]. A fundamental challenge lies in achieving stable signal acquisition and efficient energy transduction under complex mechanical deformations, while simultaneously fulfilling critical requirements of biocompatibility, environmental tolerance, and long-term operational reliability [4,5]. Conventional rigid electronic materials (e.g., silicon wafers, metal oxides) suffer from intrinsic mechanical limitations (typical bending strength < 0.3% strain) in conforming to dynamically curved biological surfaces, while elastomer- or textile-based flexible substrates often encounter insufficient conductivity and interfacial impedance mismatch [6,7,8]. These conflicting demands have driven the rapid advancement of conductive hydrogels—a class of multifunctional materials that integrate conductive media (e.g., carbon-based materials, conductive polymers, metallic nanostructures, or ionic networks) with hydrophilic polymer matrices, successfully reconciling the traditionally incompatible triad of flexibility, conductivity, and environmental stability to emerge as an ideal platform for high-performance flexible sensors [9,10,11]. Notably, biopolymer-based hydrogels (e.g., chitosan, sodium alginate, gelatin) are gaining prominence due to their inherent biocompatibility, biodegradability, and renewable sourcing, addressing growing demands for sustainable and implantable sensor technologies in healthcare and environmental applications [12,13].

The exceptional merits of conductive hydrogels stem from their tailorable multi-scale structural design and functional synergy mechanisms [9]. Recent advancements in fabrication strategies have enabled precise modulation of conductive components: carbon-based materials (graphene, carbon nanotubes (CNTs), MXene) establish three-dimensional conductive networks through their high specific surface areas and electron mobility [14,15,16]; conductive polymers (poly(3,4-ethylenedioxythiophene):polystyrene sulfonate (PEDOT:PSS), polyaniline (PANI)) achieve efficient charge transport via π-conjugated molecular chains [17,18,19]; metallic systems (silver nanowire (AgNW), liquid metals (LMs)) combine intrinsic metallic conductivity with dynamic topological structures to optimize interfacial responsiveness [20]; while ionic hydrogels leverage reversible ion migration mechanisms to attain self-healing capability and environmental adaptability [21,22,23,24]. Biopolymer matrices further enhance these systems by introducing stimuli-responsive functionalities and enabling enzyme-mediated signal transduction for biochemical sensing [25]. Crucially, multicomponent hybridization strategies (e.g., carbon–polymer composites, metal-ion synergies) further enhance material performance through interface engineering and cross-scale coupling effects [18,26,27]. Hydrogels often exhibit temperature-responsive behavior due to phase transitions in the polymer network, where elevated temperatures may accelerate water evaporation and lead to shrinkage, while lower temperatures enhance water retention but may affect the mechanical stability [28]. Changes in ambient humidity can significantly influence the swelling and deswelling dynamics, which directly impacts the hydrogel’s mechanical and electrical properties, especially in flexible sensing applications [29]. Furthermore, under continuous mechanical stress, hydrogels may experience structural fatigue, reduced elasticity, or micro-fracturing, which can compromise their long-term durability [30]. To overcome these challenges, strategies such as incorporating double-network structures or reinforcing nanomaterials have been shown to enhance mechanical robustness [30]. The integration of biopolymers with synthetic conductive networks has yielded “green” hybrid hydrogels that combine natural biocompatibility with engineered electrical properties, opening avenues for eco-friendly sensors and biodegradable diagnostic platforms [31]. This structure–function integrated design philosophy empowers conductive hydrogels to address diverse sensing requirements spanning from microstrain detection to biomolecular recognition.

**Figure 1:**
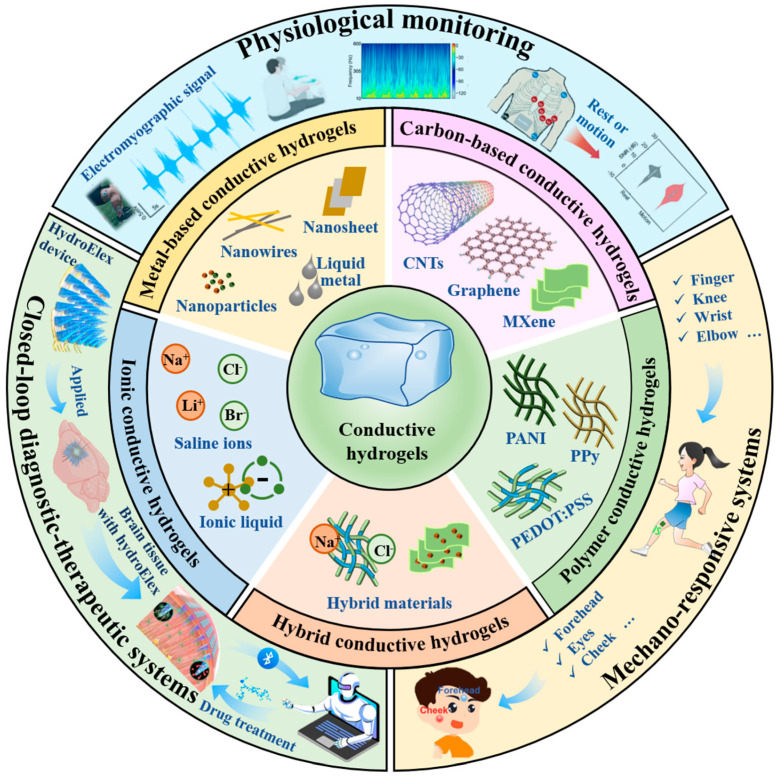
Overview of types, design strategies, and emerging applications of conductive hydrogels in wearable sensing. This figure is original, with some elements modified from copyrighted pictures that were used with permission.

This review outlines design and performance optimization strategies for conductive hydrogels (metal-, carbon-, polymer-, ionic-, and hybrid-based), focusing on three key aspects: conductive network architecture, interfacial reinforcement, and environmental resilience under varying operational conditions. Wearable sensors based on these functional hydrogels have been widely applied in real-time physiological signal monitoring, mechano-responsive sensing, and closed-loop diagnostic–therapeutic systems, providing important technological support for non-invasive health monitoring and intelligent diagnostics (Figure 1). This analytical framework not only provides theoretical foundations for developing high-performance flexible sensors but also accelerates the translational progression of conductive hydrogels from laboratory prototypes to industrial-scale applications, ultimately bridging the gap between advanced material innovation and real-world technological implementation.

## 2. Type and Design Strategies of Conductive Hydrogels

Conductive hydrogels, as an advanced material system integrating flexible matrices with conductive functionalities, have emerged as a prominent candidate in wearable sensing technologies due to their exceptional scenario adaptability and superior signal sensitivity [32]. The fundamental design philosophy centers on the synergistic engineering of conductive fillers and hydrophilic polymer networks to achieve efficient electrical signal transduction from diverse physical stimuli, including strain, pressure, temperature, and humidity [9,21]. From the perspective of material classification, conductive hydrogel sensors are systematically categorized into several types based on their conductive components: metal-based systems, carbon-based systems, conductive polymer-based systems, ionically conductive systems, and hybrid conductive systems incorporating mixed conductive materials [33]. This classification framework not only elucidates the compositional diversity of conductive hydrogels but also underscores the critical design principle of tailoring conductive media selection and network architecture modulation (e.g., gradient distribution, porous engineering) for performance-oriented optimization. Such strategic material engineering establishes a robust foundation for their multifunctional applications in health monitoring, human–machine interfaces, and intelligent robotics, driving innovations in next-generation flexible electronics.

### 2.1. Metal-Based Conductive Hydrogels for Wearable Sensors

Metal-based conductive hydrogels (e.g., AgNW, LM, metallic nanoparticles) have emerged as a transformative platform in flexible sensing technologies by synergistically integrating the intrinsic advantages of inherent metallic conductivity and mechanical compliance [34]. These materials exhibit multimodal signal perception and robust environmental tolerance, enabling reliable operation under complex sensing scenarios [35]. The functional design strategies of metal-incorporated hydrogels can be systematically categorized into two distinct architectures: (1) metal nanoparticle/nanowire-embedded composites (e.g., silver nanoparticles, gold nanowires), where in situ reduction or chemical bonding establishes continuous conductive pathways within hydrogel networks, achieving balanced conductivity and dynamic stress dissipation [36,37]; and (2) LM-integrated systems (e.g., gallium-based alloys), leveraging fluidic behavior to create adaptive conductive interfaces that enable ultra-stretchability and instantaneous crack repair, with pressure-responsive thresholds tunable through microchannel engineering [38,39]. By synergistically integrating metal nanostructures with hydrogel matrices, metal-based conductive hydrogels achieve unparalleled conductivity, mechanical durability, and interfacial adaptability, enabling ultra-sensitive biosignal monitoring through low-impedance skin coupling, stable operation in extreme environments (e.g., sub-zero temperatures or high humidity via dynamic metal–polymer interactions), and long-term wearability via fatigue-resistant networks. These advancements address critical limitations of carbon-, polymer-, or ion-based counterparts, such as conductivity degradation, signal drift, and environmental instability, establishing metal-based systems as transformative platforms for next-generation intelligent sensing systems [34,40].

**Figure 2:**
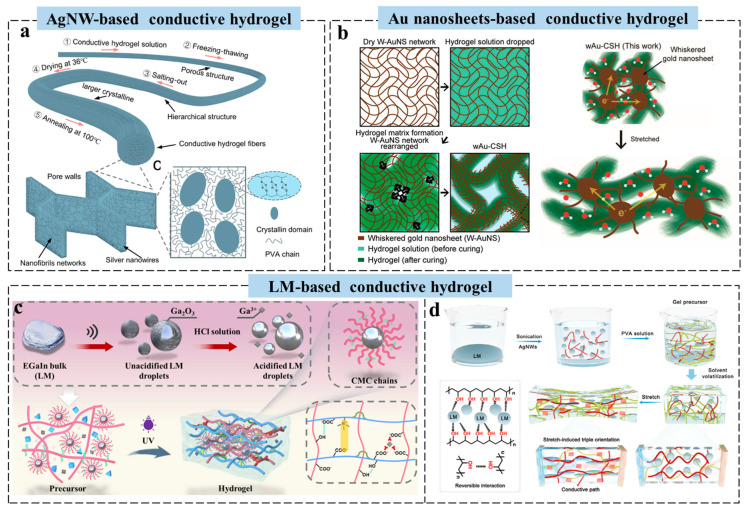
Design strategy of metal-based conductive hydrogels for wearable sensors. (**a**) Schematic illustration of the preparation of conductive hydrogel fibers [41]. (**b**) Schematic illustrations of the sequential formation method for stretchable hydrogel nanocomposites [42]. (**c**) Schematic illustration of the preparation of an LM-based conductive hydrogel [45]. (**d**) Schematic illustration of the preparation process and stretch-induced triple orientation in the PAL hydrogels [50]. All pictures have adopted with permission.

Recently, Wang et al. developed a strategy to fabricate conductive hydrogel fibers with high conductivity, enhanced water stability, excellent mechanical properties, and fatigue resistance by constructing AgNW-reinforced polyvinyl alcohol (PVA) networks (Figure 2a). Through the combined effects of freeze-thawing, salting-out, and dry-annealing, continuous phase separation induces hierarchical structures, which promote interconnected conductive networks and enhance fiber crystallinity. These fibers can be processed into yarns and fabrics, demonstrating strong potential for bioelectronic applications [41]. Moreover, a highly conductive and stretchable hydrogel nanocomposite was developed by integrating whiskered Au nanosheets into hydrogel matrices (Figure 2b). The pre-formed dry gold network maintains tight interconnections within the hydrogel, enabling a stable percolation network even under large deformations. The gold–hydrogel nanocomposites achieve conductivity up to ~520 S cm⁻^1^ with stretchability around 300%, without requiring dehydration. By tuning the density of the gold network, conductivity can be further increased to ~3300 S cm⁻^1^ [42].

While metallic nanomaterials enhance hydrogel conductivity and mechanics, modulus mismatch between rigid nanofillers and the soft matrix induces interfacial stress concentration, microcrack propagation, and irreversible delamination during cyclic deformation, limiting long-term reliability in dynamic applications such as wearable sensors [43,44]. In contrast, LMs, with comparable modulus, offer superior conductivity, fluidity, and processability. An oxide-free LM-doped double network hydrogel (carboxymethyl cellulose (CMC)/poly (acrylic acid-co-acryloyloxyethyltrimethyl ammonium chloride) P(AA-co-DAC)/NaCl-LM) was prepared using CMC and functionalized CMC as the first network, and P(AA-co-DAC) as the second network. Synergistic interactions between the networks impart excellent mechanical properties, high conductivity, and stable performance (Figure 2c). A triboelectric nanogenerator (TENG) based on this hydrogel further demonstrated excellent electrical output and high pressure sensitivity [45]. Eutectic gallium–indium alloy (EGaIn) is another LM with high conductivity, environmental stability, and deformability, commonly incorporated into hydrogels to enhance toughness [46,47,48]. However, poor compatibility with polymers often leads to leakage and limited conductivity due to incomplete conductive pathways [49]. To address this, Wang et al. developed PVA-AgNW-LM (PAL) hydrogels, where stretch-induced orientation of AgNW, deformable LM, and PVA nanocrystals synergistically enhance mechanical properties (fracture stress ~30 MPa, strain >5000%) and conductivity (~24 S m⁻^1^) (Figure 2d). The LM aligns with AgNW under strain, forming efficient conductive networks. Tuning LM content enables strain-insensitive resistance or positive piezoconductivity, with conductivity increasing up to 6000 times under strain. The oriented nanocrystals, LM, and AgNW enhance crack resistance, fatigue durability, and recyclability in water [50]. However, while metallic nanoparticles endow hydrogels with enhanced conductivity, their practical implementation in bioelectronics faces critical challenges that demand thorough evaluation [35,40]. Beyond the inherent limitations of nanoparticle aggregation and compromised polymer compatibility, the long-term stability of conductive networks is jeopardized by oxidative degradation mechanisms in physiological environments [36,38,39]. Furthermore, biocompatibility concerns persist due to the potential leaching of toxic metal ions and insufficient understanding of nanoparticle–cell interactions over extended durations [44]. These unresolved material safety issues, coupled with the reliance on costly noble metal precursors, collectively hinder the scalability and clinical translation of metal-based conductive hydrogels.

### 2.2. Carbon-Based Conductive Hydrogels for Wearable Sensors

Carbon-based conductive hydrogels (e.g., graphene, MXene, CNTs) leverage high specific surface areas and percolation networks to enhance charge transport dynamics, while exhibiting exceptional mechanical flexibility and environmental stability, making them ideal for multifunctional sensing platforms [10,51]. Carbon-based conductive hydrogels are categorized by their carbonaceous components, each offering distinct advantages for targeted applications: (1) Graphene-based hydrogels leverage high electron mobility and interconnected networks for ultra-sensitive strain/pressure sensing (e.g., epidermal pulse monitoring) [52]; (2) CNT-embedded hydrogels utilize mechanical robustness and stress-responsive pathways for dynamic motion sensing (e.g., joint movement tracking) [53]; and (3) MXene-integrated hydrogels exploit hydrophilic surface groups for humidity-responsive and self-healing sensors (e.g., moist wound monitoring) [54,55]. Hybrid systems (e.g., graphene-CNTs) further enhance charge transfer and durability through synergistic interactions, enabling multifunctional sensing under harsh conditions [56,57].

**Figure 3:**
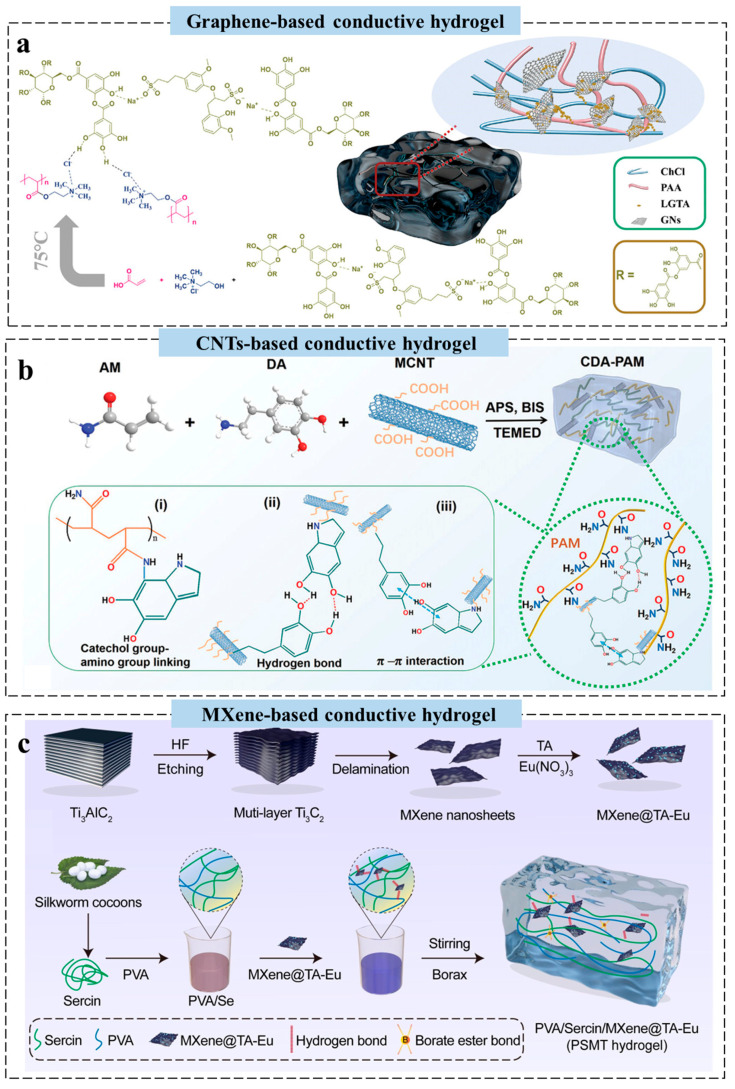
Design strategy of carbon-based conductive hydrogels for wearable sensors. (**a**) The synthesis mechanism of a graphene-based conductive hydrogel [52]. (**b**) Schematic diagram of the preparation process of a CNT-based conductive hydrogel [51]. (**c**) Schematic diagram of the synthesis of an MXene-based conductive hydrogel [55]. All pictures have adopted with permission.

Graphene, a two-dimensional monolayer of carbon atoms arranged in a hexagonal honeycomb lattice, has emerged as a highly promising material for enhancing hydrogel performance due to its exceptional electrical conductivity, remarkable mechanical strength, and superior thermal stability [58]. Wang et al. developed a multifunctional lignin–tannin nanosphere/graphene-doped hydrogel (LTGH) as a wearable flexible pressure sensor (Figure 3a). Self-assembled sodium lignosulfonate and tannic acid nanospheres effectively dispersed graphene within the hydrogel, ensuring uniform distribution and significantly improving its properties. The resulting LTGH demonstrated excellent electrical conductivity, a wide linear sensing range, high sensitivity, strong adhesion, an ultra-low detection limit, UV resistance, and antibacterial performance [52]. This bio-inspired dispersion strategy eliminates the need for toxic solvents, aligning with scalable and sustainable manufacturing principles. However, the reliance on CVD-derived graphene in this study highlights a cost bottleneck; replacing it with cost-effective graphene oxide derivatives could enhance scalability, albeit at a trade-off in conductivity [59].

CNTs, with excellent conductivity, flexibility, and high specific surface area, enhance charge transfer efficiency, mechanical strength, and multifunctional sensing capabilities in conductive hydrogels [60]. Recently, a flexible, self-powered TENG wound patch (e-patch) was developed to promote wound healing through combined electrostimulation and photothermal effects. The e-patch was fabricated using a polyacrylamide/polydopamine (PAM/PDA) dual-network hydrogel doped with multi-walled CNTs, offering high conductivity, stretchability, and biocompatibility, while enabling real-time detection of mechanical and electrical signals during human motion (Figure 3b) [61]. While the solvent-casting method used here is compatible with roll-to-roll production, the incorporation of CNTs via sonication limits throughput. Emerging 3D printing and bio-templated dispersion strategies, leveraging self-assembled biomolecular frameworks, enable scalable and surfactant-free CNT integration in hydrogels while preserving dispersion homogeneity, thereby aligning with green manufacturing paradigms [62].

MXene, a novel two-dimensional nanomaterial, is widely applied in biomedical fields due to its excellent biocompatibility, photothermal conversion, and electrical conductivity [63]. Zhang et al. developed a composite hydrogel to promote infected wound healing via near-infrared and electrical stimulation therapy. MXene nanosheets modified with a TA-Eu metal-phenolic network (MPN) were incorporated into a dynamic hydrogel network of natural sericin, PVA, and borax, imparting shape plasticity, self-healing, and strong adhesion (Figure 3c). The hydrogel reduced inflammation through photothermal antimicrobial effects and oxidative stress elimination, while its high conductivity promoted fibroblast proliferation and migration under electrical stimulation therapy [55]. Beyond spray-coating, emerging alternatives such as electrostatic self-assembly and roll-to-roll dip-coating could eliminate vacuum dependencies, reduce energy consumption, and enhance automation compatibility, albeit requiring optimization of MXene suspension rheology and interfacial adhesion [64]. Nevertheless, carbon-based conductive hydrogels often suffer from heterogeneous conductivity due to uneven dispersion of conductive fillers [65]. Under dynamic mechanical loading, the conductive network is prone to fracture, compromising signal stability. Additionally, high filler content may adversely affect the intrinsic flexibility of the hydrogel matrix.

The industrial translation of high-performance carbon-based conductive hydrogels demands urgent attention to scalability barriers, including sustainable 2D material synthesis, standardized dispersion protocols, and automation-driven fabrication (e.g., microfluidic molding) [66]. To bridge the lab-to-industry gap, future studies should rigorously quantify scalability metrics, such as material costs, energy consumption, and batch consistency, in parallel with functional performance evaluations.

### 2.3. Conductive Polymer-Based Hydrogels for Wearable Sensors

Conductive polymer-based hydrogels (e.g., PANI, polypyrrole(PPy), PEDOT:PSS), relying on π-π conjugated molecular architectures and adjustable doping mechanisms to achieve high sensitivity, mechanical flexibility, and environmental robustness, demonstrate exceptional potential in flexible sensing technologies [32]. Unlike traditional conductive systems (e.g., metals or carbon-based materials), these polymers enable programmable molecular customization through backbone engineering (e.g., optimizing conjugation length for enhanced conductivity), side-chain modification (e.g., grafting functional groups for improved hydrophilicity or stability), and composite hybridization (e.g., integrating nanomaterials for synergistic charge transport), allowing precise tailoring of electrochemical and mechanical properties to meet specific sensing requirements [67]. PANI-based hydrogels capitalize on reversible proton doping mechanisms to develop pH-responsive biosensing platforms, often integrated with biocompatible polymers like polyvinyl alcohol for physiological biomarker detection [68]. PPy-derived systems employ in situ polymerization strategies to enhance charge transport efficiency, while their hybridization with carbon nanomaterials improves mechanical resilience for dynamic motion sensing [69,70]. PEDOT:PSS-based hydrogels utilize inherent conductivity and aqueous compatibility to create dual electron–ion conductive networks, achieving temperature-adaptive operation and self-repairing functionalities for extreme-environment sensing and epidermal electronics [71].

**Figure 4:**
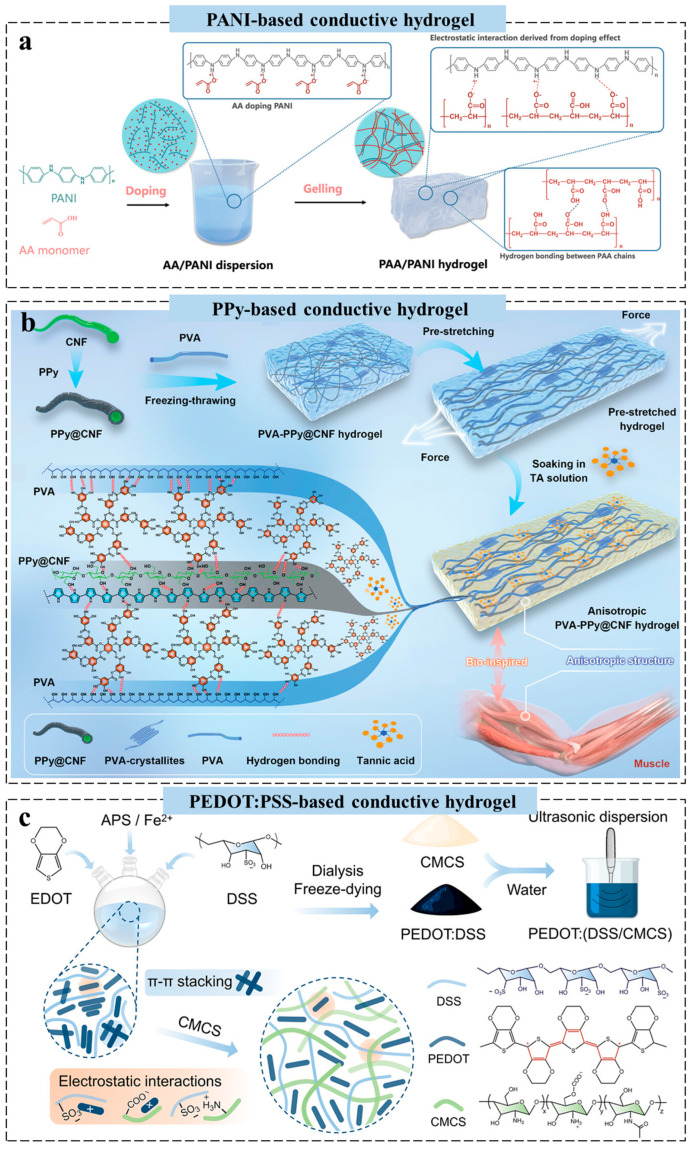
Design strategy of conductive polymer-based conductive hydrogels for wearable sensors. (**a**) Schematic illustration of the synthesis process for a PANI-based conductive hydrogel [72]. (**b**) Schematic diagram for the preparation of anisotropic PPy-based conductive hydrogels and their possible formation mechanism [73]. (**c**) Schematic diagram for the preparation of an anisotropic PEDOT:PSS-based conductive hydrogel [76]. All pictures have adopted with permission.

Recently, a “doping-then-gelling” strategy was proposed to synthesize a supramolecular PANI/PAA hydrogel with a specific strand-entangled network, where acrylic acid monomers were used to dope PANI and prevent its aggregation (Figure 4a). The strong electrostatic interactions between PAA and PANI chains acted as dynamic bonds, driving the formation of the entangled structure, which endowed the hydrogel with excellent stretchability, high strength, and rapid self-healing ability. Furthermore, a PAA/PANI hydrogel-based sensor was developed, exhibiting high strain sensitivity, fast response, and stable conductivity under cyclic stretching [72]. Traditional isotropic conductive hydrogels often exhibit poor mechanical properties and limited sensing performance due to insufficient reinforcement and energy dissipation mechanisms, hindering their use in wearable electronics. Rational structural design is thus critical to enhance mechanical strength and achieve anisotropic functionality [73]. Inspired by human muscle structures, Lin et al. developed a robust anisotropic conductive hydrogel by aligning polyvinyl alcohol and PPy-decorated cellulose nanofibrils, followed by tannic acid crosslinking (Figure 4b). TA locks the anisotropic structure through multiple hydrogen bonds, imparting excellent mechanical properties, anisotropic adhesion, and direction-dependent conductivity. The resulting hydrogel strain sensor enables stable monitoring of complex joint movements and control of a multiaxial virtual robot manipulator [73].

Moreover, among conductive hydrogels, PEDOT:PSS has attracted attention among conductive hydrogels for its high conductivity, tunability, and commercial availability [74]. Its conductivity can be tuned by adjusting PEDOT’s oxidation state and molecular conformation. In PEDOT:PSS hydrogels, PEDOT provides conductivity, PSS ensures water solubility and stability, and water forms the hydrogel matrix [75]. Yu et al. proposed a multilevel template dispersion strategy to fabricate PEDOT:(DSS/CMCS) with enhanced biocompatibility compared to commercial PEDOT:PSS, while maintaining good processability and conductivity. Combined with oxidized dextran, PEDOT:(DSS/CMCS) forms an injectable hydrogel via dynamic covalent imine bonds under mild conditions (Figure 4c). The hydrogel features tissue-matched modulus and conductivity, enabling adaptation to dynamic tissue environments and mitigating fibrosis-induced electrical decoupling [76]. However, irreversible oxidation and residual organic solvents from processing pose risks to long-term stability and biocompatibility, hindering their reliability in dynamic biointerfaces.

### 2.4. Ionic Conductive Hydrogels for Wearable Sensors

Ionic conductive hydrogels (e.g., saline solutions, ionic liquids, dynamic ion-crosslinked networks), operating through mobile ion migration under external stimuli, offer unique advantages in sensing applications [77]. Their soft nature enables conformal contact with dynamic biological surfaces (e.g., skin, joints) without mechanical mismatch, while supporting multimodal signal readouts [78]. These features establish them as adaptive, biocompatible platforms for next-generation wearable sensors and real-time health monitoring systems.

**Figure 5:**
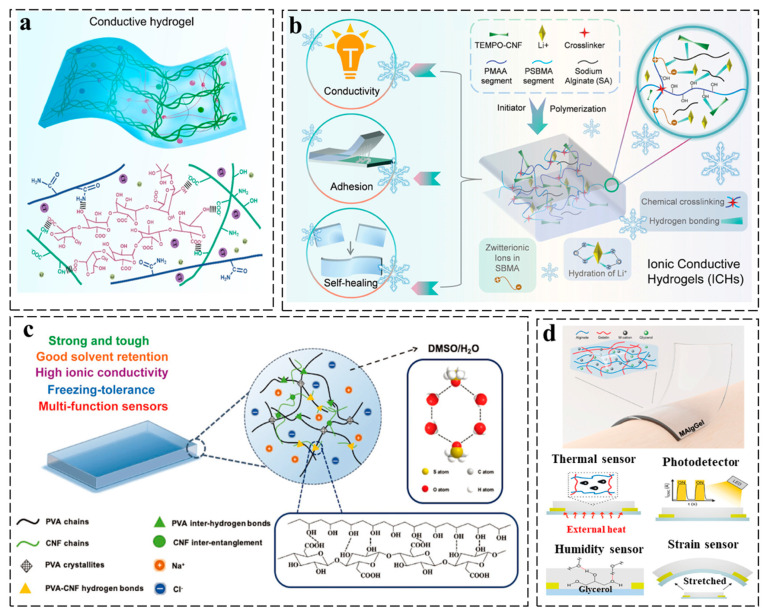
Design strategy of ionic conductive hydrogels for wearable sensors. (**a**) The ionic conductive hydrogel and its network structure [79]. (**b**) Schematic illustration of the synthesis and properties of an ionic conductive hydrogel [82]. (**c**) Schematic illustration of a PVA-CNF organohydrogel [83]. (**d**) Three-dimensional schematic of a metal-crosslinked alginate/gelatin organohydrogel (MAlgGel) conformally attached to skin for sensing [85]. All pictures have adopted with permission.

For instance, Li et al. developed a body temperature-responsive adhesive ionic conductive hydrogel by integrating PAM, gelatin, and sodium alginate (Figure 5a). The gelatin-mediated thermoresponsive behavior enables rapid adhesion enhancement at body temperature and painless detachment upon cooling within seconds. With a skin-like Young’s modulus, the hydrogel achieves conformal skin contact and low electrode-epidermal impedance, facilitating high-fidelity bioelectrical signal recording and precise motion detection [79]. Despite their exceptional properties, ionic conductive hydrogels encounter significant challenges in wearable and flexible electronics owing to their inherent temperature sensitivity. Specifically, at elevated temperatures, water evaporation leads to dehydration of the hydrogel network, whereas under low-temperature conditions, freezing results in ice crystallization. These thermally induced phase changes severely degrade ionic conductivity and mechanical stability [80,81]. These limitations hinder their practical deployment in real-world environments. Han et al. developed an anti-freezing ionic conductive hydrogel by integrating sulfobetaine methacrylate, methacrylic acid, TEMPO-oxidized cellulose nanofibers, sodium alginate, and lithium chloride into a dual-crosslinked network (Figure 5b). This design achieves high conductivity, strong adhesion, and self-healing capability even at −80 °C, with sustained functionality for over 45 days. The hydrogel enables conformal skin attachment and high-fidelity signal transmission, making it suitable for non-invasive health monitoring and human–machine interaction in extreme environments, including cryogenic and space applications [82]. Moreover, an anti-freezing ionic conductive organohydrogel was engineered through polyvinyl alcohol/cellulose nanofibril (CNF) assembly in a DMSO-water system, achieving unprecedented mechanical–electrical synergy with high stretchability, strength, and ionic conductivity (Figure 5c). The CNF-reinforced network uniquely resolves the intrinsic conflict between mechanical robustness and ion transport efficiency while maintaining flexibility and conductivity at −70 °C. This freeze-resistant design enables sensitive strain/pressure detection with exceptional environmental adaptability, demonstrating potential for extreme-condition motion sensors in wearable and biomedical applications [83].

Ionic conductive hydrogels for multimodal monitoring leverage their unique ion transport mechanisms and intrinsic flexibility to simultaneously detect diverse physiological signals, offering an integrated platform for real-time, precise health monitoring [84]. Tordi et al. engineered alginate–gelatin organohydrogels with visible-light transparency, ionic conductivity, and multimodal responsiveness through metal cation-mediated crosslinking (Figure 5d). This strategy enables precise tuning of thermal/mechanical properties while achieving environmental sensing capabilities for temperature, humidity, and strain variations. The material’s multistimuli-responsive behavior demonstrates potential for real-time monitoring systems in environmental and wearable health applications [85]. Ionic conductive hydrogels, however, face challenges such as environmental sensitivity and limited long-term stability due to electrolyte leakage or mechanical fatigue under cyclic deformation, which may compromise their reliability in practical applications [86]. Additionally, their lower conductivity compared to electronic counterparts restricts signal response speed and resolution in high-demand sensing scenarios.

### 2.5. Hybrid Conductive Hydrogels for Wearable Sensors

Hybrid conductive hydrogels integrate diverse conductive components (e.g., conductive polymers, carbon nanomaterials, metal nanoparticles, and ionic conductors) into hydrogel matrices to optimize interfacial charge transfer efficiency, synergistically enhancing electrical conductivity, mechanical robustness, and multifunctionality for advanced sensing applications [87]. By leveraging multimodal complementary interactions—such as hydrogen bonding (enabling dynamic self-healing), π-π stacking (enhancing electron delocalization), and ionic crosslinking (regulating ion migration)—these hybrids overcome limitations of single-component systems (e.g., brittleness, interfacial mismatch, or environmental instability), achieving tunable conductivity, high stretchability, and humidity-resistant stability [87,88]. Their hybrid design enables multimodal sensing (e.g., strain, pressure, biochemical signals) through coupled electronic–ionic conduction mechanisms, while maintaining conformal contact with dynamic biological interfaces via minimized mechanical mismatch [88].

**Figure 6:**
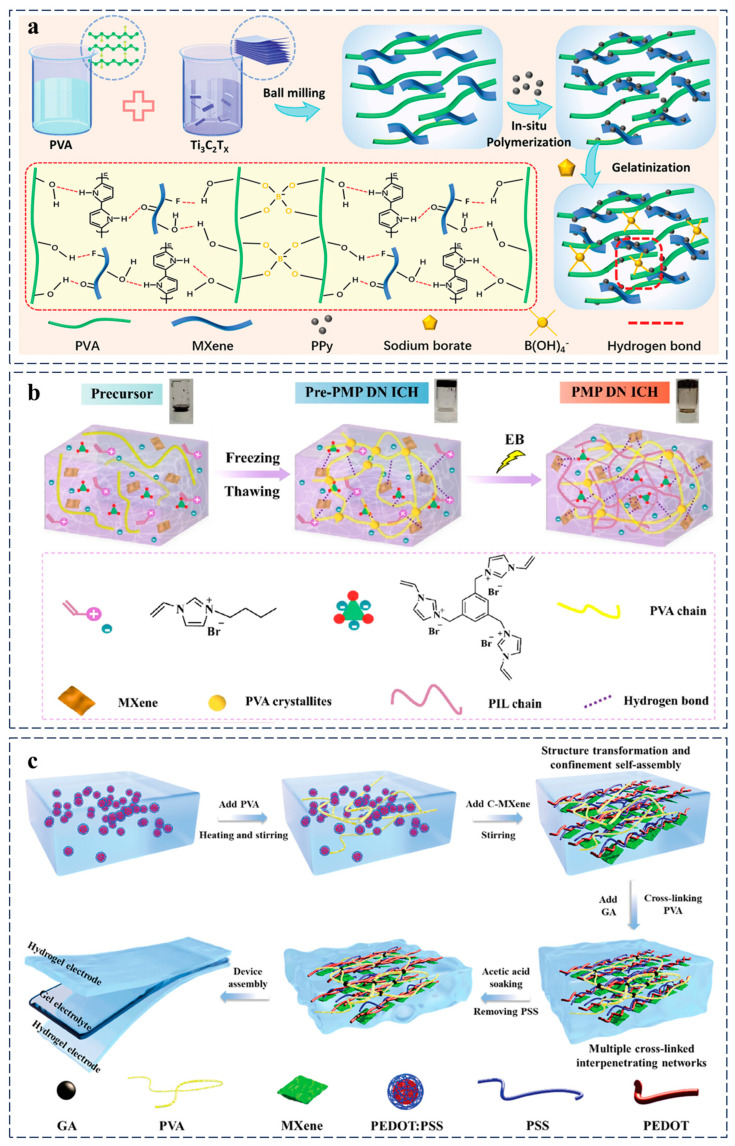
Design strategy of hybrid conductive hydrogels for wearable sensord. (**a**) Schematic of the PMP hydrogel preparation process, highlighting chemical crosslinking sites within the conductive network [89]. (**b**) Schematic of the PIL/MXene/PVA double-network ion-conducting hydrogel construction [90]. (**c**) Schematic diagram of the preparation procedures for organic–inorganic hybrid conductive hydrogels and all-hydrogel supercapacitors [87]. All pictures have adopted with permission.

Qin et al. developed an ultra-tough conductive hydrogel (PMP) via synergistic integration of PVA, MXene nanosheets, and PPy, where MXene/Fe^3^⁺ dual crosslinkers enable simultaneous enhancement of conductivity and mechanical stretchability (>4000% strain) (Figure 6a). The dynamic borate ester bonds confer rapid self-healing and repeatable adhesion, while the hierarchical network architecture facilitates strain-sensitive capacitive sensing. This design achieves precise monitoring of physiological activities, including joint motion, pulse waves, and facial micro-expressions, demonstrating potential for next-generation adaptive bioelectronics [89]. Moreover, a radiation-assisted freeze–thaw strategy was proposed to fabricate a poly(ionic liquid) (PIL)/MXene/PVA double-network hydrogel with multifunctional integration (Figure 6b). The synergistic design achieves high ionic conductivity (63.89 mS cm⁻^1^), broad thermal tolerance (−60–80 °C), long-term stability, and antibacterial activity through reinforced interfacial interactions. Demonstrating exceptional environmental adaptability, the hydrogel enables multifunctional applications including strain/temperature sensing, energy storage, and self-powered systems, establishing a robust platform for integrated soft electronics with reliable signal transmission capabilities [90].

Achieving ideal microstructures in conductive polymer hydrogels that balance stress dissipation with uninterrupted conductive pathways remains a significant design challenge. Cheng et al. developed an innovative organic–inorganic hybrid conductive hydrogel system via a synergistic confinement self-assembly and multi-crosslinking strategy. By integrating conductive PEDOT:PSS, carboxyl-functionalized titanium carbide (C-MXene), and glutaraldehyde-crosslinked PVA, the methodology facilitated the formation of a biphasic interpenetrating crosslinked network. This dual-network architecture enabled structural reconfiguration along the stretching direction under mechanical stress, significantly enhancing mechanical robustness while maintaining uninterrupted connectivity of the entangled conductive phase (PEDOT:PSS/C-MXene). The confinement-driven assembly ensured uniform dispersion of inorganic components, whereas multi-crosslinking (covalent and dynamic bonds) stabilized the hybrid interface, synergistically addressing the conductivity–flexibility trade-off in conventional hydrogels (Figure 6c). This advanced material system demonstrates unprecedented performance by simultaneously integrating high conductivity (2000 S m^−1^), substantial stretchability (200%), and superior electrochemical activity, surpassing conventional conductive hydrogel counterparts [87]. Hybrid conductive hydrogels are constrained by multicomponent processing complexity and interfacial incompatibility, which can induce mechanical/electrical fatigue during cyclic operation. Furthermore, biocompatibility concerns arising from residual components (e.g., solvents, metal ions) may undermine their reliability in biomedical deployment.

### 2.6. Analysis of Limitations and Challenges in Conductive Hydrogels

Despite their promise in flexible electronics and biomedicine, the practical application of conductive hydrogels is constrained by several challenges. A performance comparison of different types of conductive hydrogels is shown in Table 1.

Metal-based hydrogels exhibit excellent conductivity and mechanical properties but still face significant limitations. Noble metals (e.g., gold, silver) offer inherent biocompatibility, but liquid metals (e.g., gallium-based alloys) require surface coatings to mitigate toxicity risks [38,39]. Uncoated metal nanoparticles, such as copper, degrade over time due to oxidation or ion release, causing localized toxicity, chronic inflammation, or even organ damage (e.g., liver/kidney accumulation) [36,37]. Repeated mechanical deformation exacerbates interfacial delamination between metals and polymer matrices, accelerating particle migration into tissues and triggering foreign-body reactions. These issues, combined with the high cost of noble metals, limit their scalability for wearable or implantable devices.

Carbon-based hydrogels achieve high conductivity through graphene or carbon nanotubes but suffer from intrinsic brittleness and agglomeration-induced conductivity loss [62]. Unfunctionalized carbon materials provoke inflammatory responses due to hydrophobic surfaces and ROS generation, while surface modifications to enhance biocompatibility often sacrifice conductivity [65]. Long-term wearable device applications risk shedding non-degradable carbon nanotube fragments, which persist in biological systems and may trigger chronic inflammation, tissue fibrosis, or necessitate removal interventions, thus compromising safe skin integration and wound interface compatibility [91].

Conductive polymer-based hydrogels (e.g., PEDOT:PSS, polypyrrole) balance cost and tunable conductivity but degrade under moisture, heat, or UV exposure, releasing cytotoxic redox byproducts (e.g., polyaniline oligomers) [75]. Their moderate mechanical strength limits high-load applications, and prolonged electrical stimulation accelerates polymer degradation, further leaching harmful residues. Blending with biocompatible polymers (e.g., gelatin) improves safety but complicates design and compromises performance [75].

Ionic hydrogels excel in biocompatibility by mimicking biological ionic environments, yet their low conductivity (1–3 orders below electronic conductors) restricts high-frequency applications [77]. Hydration-dependent performance poses risks in dry environments: dehydration-induced shrinkage mechanically irritates tissues, while ion leakage disrupts local electrophysiology (e.g., neural signaling). Although natural polymer-based variants (e.g., alginate) support tissue integration, mismatched degradation rates may cause premature structural collapse in long-term implants [92].

Hybrid hydrogels aim to synergize material advantages but face interfacial incompatibility. Weak metal–polymer bonding promotes particle release and catalytic degradation, while carbon–ionic conductor hybrids risk charge imbalance-induced polarization. Multi-component systems also amplify toxicity risks (e.g., metal ions and polymer fragments) and provoke complex immune responses. Scalability remains hindered by high costs and unpredictable interactions, undermining their potential for multifunctional implants [87].

### 2.7. Environmental and Ethical Considerations of Conductive Hydrogels

The increasing use of conductive hydrogels in wearable electronics and biomedical applications has raised concerns about their environmental footprint and ethical implications [93]. A critical aspect to consider is the energy-intensive synthesis processes involved in the production of certain conductive hydrogel materials. For instance, polymerization reactions, chemical crosslinking, and the incorporation of conductive fillers (such as carbon nanomaterials or metal nanoparticles) often require high energy inputs and the use of organic solvents or toxic reagents, contributing to environmental pollution and posing potential health risks [94].

Disposal and end-of-life management of conductive hydrogels is another area that warrants attention. Traditional synthetic hydrogels, particularly those incorporating non-biodegradable polymers, may persist in the environment for extended periods, leading to long-term ecological impacts. Moreover, the recycling of hybrid hydrogels containing multiple functional components remains challenging due to the complexity of their structures [95]. Without effective recycling strategies, these materials may contribute to environmental waste.

To mitigate these concerns, researchers are exploring greener synthesis approaches that utilize renewable resources, minimize the use of hazardous chemicals, and reduce energy consumption. For example, the development of bio-based conductive hydrogels derived from natural polymers such as cellulose, chitosan, or alginate has shown promise in enhancing biodegradability and reducing environmental impact [96]. Additionally, self-healing and recyclable hydrogel systems are being designed to extend the lifespan of these materials, reducing the need for frequent replacements and minimizing waste generation [97].

Ethically, the selection of materials for conductive hydrogels should also consider the potential risks associated with long-term exposure in biomedical applications [98]. Ensuring biocompatibility and minimizing cytotoxicity are essential for safeguarding human health and preventing unforeseen adverse effects. As the field advances, establishing regulatory frameworks and lifecycle assessments will be critical to guiding the responsible development and commercialization of conductive hydrogels.

## 3. Application of Conductive Hydrogels in Wearable Sensors

### 3.1. Physiological Monitoring

**Figure 7:**
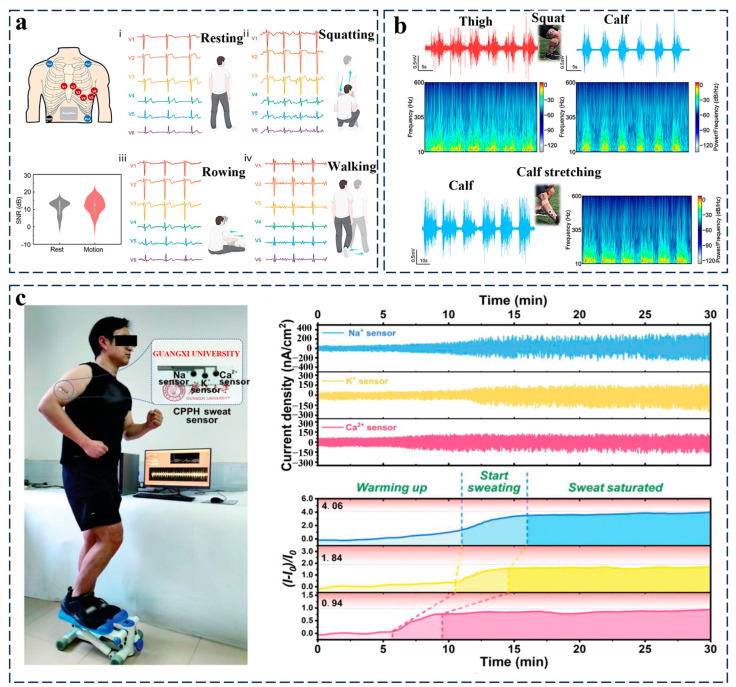
Application of conductive hydrogels in physiological monitoring. (**a**) On-skin EMG and ECG monitoring of PEDOT-based conductive hydrogels [100]. (**b**) Monitoring of EMG signals during outdoor exercise utilizing in situ biogels [101]. (**c**) Real-time monitoring of ions using the sweat sensor, coupled with wireless transmission capabilities [104]. All pictures have adopted with permission.

In the realm of physiological monitoring, conductive hydrogels exhibit distinctive capabilities in both bioelectrical signal acquisition and metabolite tracking [99]. For bioelectrical sensing applications (e.g., electrocardiography/electromyography), their tissue-mimetic elastic modulus and tunable ionic conductivity enable conformal epidermal coupling, effectively suppressing motion artifacts and ensuring high-fidelity signal acquisition during dynamic physiological activities [100]. For instance, Lao et al. engineered a bioinspired adhesive hydrogel via molecular integration of catechol motifs into a PEDOT matrix, achieving nanoscale uniformity and strain-tolerant conduction (Figure 7a). This design bridges the mechanical/electrical gap between rigid electronics and soft tissues, enabling noise-resistant biosignal capture during movement. Demonstrated through electromyography, electrocardiography, and neural interfacing under stress, the hydrogel redefines bioelectronic interfaces, advancing precision wearable diagnostics with enhanced biocompatibility [100]. Moreover, Li et al. developed a thermoresponsive biogel with dynamic liquid–solid phase transition capabilities, enabling rapid in situ interfacial adaptation through temperature-activated curing (Figure 7b). Its ultra-low interfacial impedance facilitates high-fidelity biosignal acquisition under physiological stress, demonstrated in dynamic biomechanical monitoring, muscle recovery assessment, and cardiac electrophysiological tracking. This phase-transition design paradigm bridges adaptive biointerfaces and stable signal transduction, offering a transformative framework for next-generation soft electronics in precision health monitoring applications [101].

Concurrently, in metabolite monitoring, engineered porous network architectures coupled with immobilized molecular recognition elements empower selective detection of critical biomarkers (glucose, lactate, electrolytes) within biofluids through precisely designed redox-mediated or affinity-based sensing pathways [102]. For example, Arwani et al. developed an innovative wearable sensing platform utilizing a dual-conductive hydrogel architecture for in situ detection of solid-phase cutaneous biomarkers. The system employs sequential analyte dissolution–diffusion–electrochemical conversion mechanisms, enabling simultaneous monitoring of both hydrophilic (e.g., lactate) and hydrophobic (e.g., cholesterol) biomarkers without biofluid extraction. The technology establishes a paradigm-shifting approach for non-invasive biomarker surveillance through advanced interfacial engineering of solid-state biosensing systems [103]. Moreover, a self-powered sweat sensor was developed using a cellulose-PANI hydrogel, achieving autonomous ion detection (Na⁺, K⁺, Ca^2^⁺) through triboelectric sensing and wireless data transmission (Figure 7c). This innovation integrates self-healing mechanics, extreme flexibility, and environmental resilience into a single platform, demonstrating a material-driven breakthrough in continuous, non-invasive health monitoring via energy-autonomous epidermal interfaces [104].

### 3.2. Mechano-Responsive Systems

**Figure 8:**
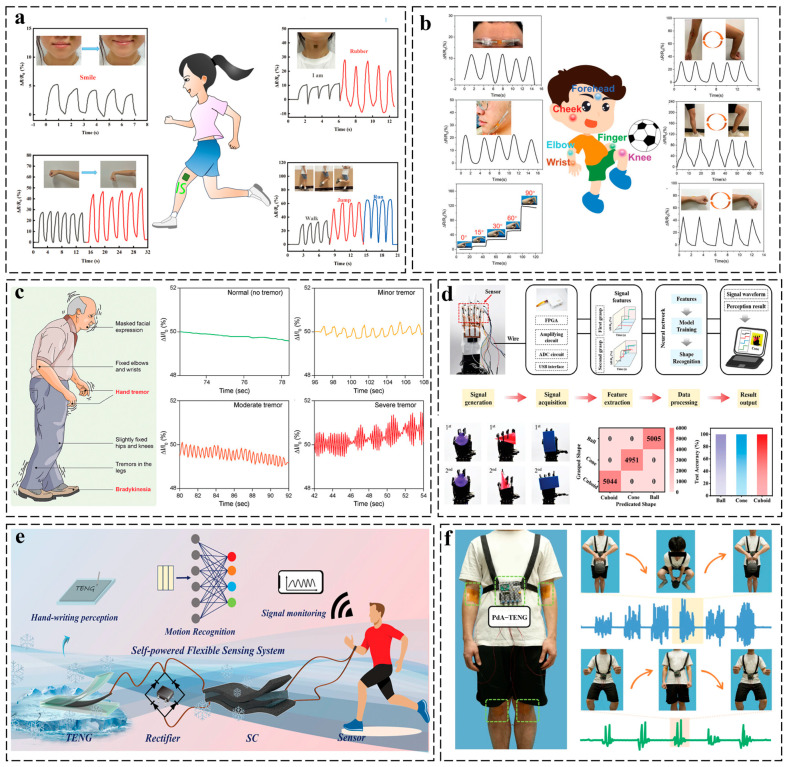
Application of conductive hydrogels in mechano-responsive systems. (**a**) Real-time monitoring of human body movements using a flexible conductive hydrogel-based sensor [17]. (**b**) Performance of the PLBOH-based strain sensor in detecting human motions [112]. (**c**) On-body monitoring of simulated Parkinson’s disease-related movement disorders and abnormal respiratory events using β-CD-g-(pAAm/pAETAc) hydrogel sensors [114]. (**d**) Machine learning-powered robotic tactility [116]. (**e**) Schematic illustration of the preparation process for the versatile PBACA-Zn^2^⁺ hydrogel and its self-powered application system [118]. (**f**) Application of PdA-TENG as a self-powered e-skin device for comprehensive physiological and motion monitoring [117]. All pictures have adopted with permission.

In wearable mechano-responsive systems, conductive hydrogels revolutionize motion sensing through tissue-matched elasticity (elastic modulus: 1–100 kPa) and strain-adaptive conductive networks with high sensitivity [105,106,107]. These materials transduce mechanical stimuli (stretching, compression, torsion) into electrical signals via polymer chain disentanglement and ion/electron redistribution mechanisms, achieving low hysteresis (<10%) and cyclic stability (>1000 cycles). Such properties enable high-fidelity monitoring of human kinematics (e.g., joint movements at 100–300% strain) and tactile interactions, while maintaining strain-invariant conductivity under dynamic conditions [108,109,110]. For instance, Huo et al. developed a bioinspired chitosan-levodopamine/polyacrylic acid hydrogel through molecular design, achieving tunable adhesion, ultra-high stretchability, and multi-stage strain sensitivity. Synergistic catechol-Zn^2^⁺ coordination enabled precise monitoring of physiological motions, from joint movements to micro-expressions, while ensuring excellent cycling stability and conductivity [111]. Our group engineered a multifunctional conductive hydrogel via radical grafting and supramolecular self-crosslinking, achieving ultra-stretchability (0–600% strain) and tissue adhesion (36.07 kPa) (Figure 8a). Integrated with flexible circuits and wireless data transmission, this closed-loop wearable platform enables real-time motion monitoring and personalized rehabilitation training [17]. Furthermore, a molecular engineering strategy synergistically combining bridge-effect-mediated polymer chain lubrication and swelling-induced entanglement elimination was developed to fabricate dynamic hydrogel networks with ultra-low hysteresis and strain–linear electromechanical response (Figure 8b). This innovative PAM/laponite/organoborax composite hydrogel demonstrates millisecond-scale stress relaxation and whole-range strain sensitivity, enabling precise detection of multiscale biomechanical signals from micro-expressions to limb movements [112].

Motor biomarkers (e.g., gait anomalies, tremors) often manifest earlier than cognitive deficits in neurodegenerative disorders like Parkinson’s disease, enabling proactive intervention and improved prognosis [113]. Roy et al. engineered a biodegradable hydrogel sensor via supramolecular pseudo-slide-ring architecture, integrating PAM with β-cyclodextrin and bioionic liquids to decouple mechano-chemical trade-offs (Figure 8c). This design achieves unprecedented synergy of mechanical resilience, conductivity, and tissue adhesion while maintaining rapid self-healing and ultra-stretchability. The sensor enables high-precision motion/haptic monitoring, particularly for early detection of neurodegenerative motor biomarkers (e.g., subclinical tremors, gait deviations), demonstrating transformative potential in pre-symptomatic disease diagnostics and sustainable personalized healthcare systems [114].

Hydrogel sensors offer soft, adhesive interfaces with multimodal tactile perception (pressure, motion, texture), positioning them as ideal platforms for biomimetic robotic somatosensory systems [115]. Chen et al. engineered a temperature-resilient organohydrogel tactile system for robotics through dynamic interpenetrating networks of PANI and poly (acrylamide-co-acrylic acid), leveraging electrostatic/hydrogen bonding interactions (Figure 8d). This design achieves ultra-low hysteresis and extreme thermal stability, while maintaining high strain/pressure sensitivity and conductivity. Integrated sensor arrays on robotic manipulators enable simultaneous proprioceptive motion tracking and object pressure mapping, with machine learning-enhanced shape recognition accuracy. The system establishes a paradigm for robust biomimetic tactile intelligence in next-generation adaptive robotics through synergistic material-engineered perception and environmental tolerance [116]. 

Furthermore, conductive hydrogels exhibit significant potential in self-powered sensing systems by enabling the direct conversion of biomechanical energy (e.g., body movements or physiological activities) into electrical signals through piezoelectric, triboelectric, or ion-gradient mechanisms, offering a groundbreaking solution for developing battery-free, energy-autonomous wearable electronics [12,117]. For instance, Wang et al. presented a topology-engineered interlocking strategy to fabricate ultra-tough hydrogels via molecular chain alignment and dual hydrogen/metal-ligand toughening. Integrating Zn²⁺-crosslinked CNFs into a PAM-poly(vinyl alcohol) network achieves exceptional mechanical strength and toughness. Mechanically aligned CNFs functionalized with polyaniline exhibit 12.7-fold higher power density through directional charge transport. A deep learning-integrated self-powered sensor demonstrated 98.2% accuracy in real-time motion recognition across 15 activities, advancing autonomous wearable systems (Figure 8e) [118]. Zhang et al. devised a cellulose nanofiber-enabled dynamic hydrogel with Fe³⁺-coordination-driven tunable adhesion and UV-triggered detachment. The hierarchical Fe³⁺-nanofiber network achieves 86% toughness modulation and 12 mS cm⁻¹ conductivity, adaptable across biopolymers and synthetic polymers. A photo-detachable triboelectric nanogenerator demonstrates real-time wireless motion monitoring via machine learning, advancing self-powered e-skins and adaptive human–machine interfaces (Figure 8f) [117].

### 3.3. Closed-Loop Diagnostic–Therapeutic Systems

**Figure 9:**
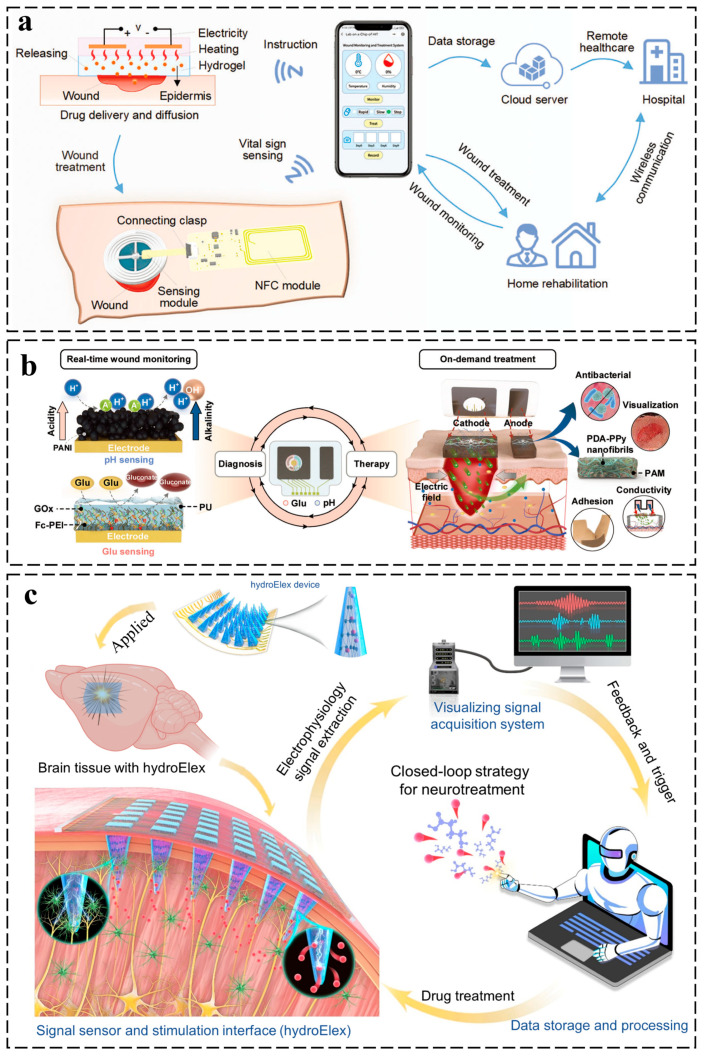
Application of conductive hydrogels in closed-loop diagnostic–therapeutic systems. (**a**) Schematic of a multifunctional IWD for exudate management and chronic wound treatment [124]. (**b**) A wireless theranostic system for diabetic wound management [125]. (**c**) Schematic diagram of a closed-loop bioelectronic system for adaptive antiepileptic treatment [127]. All pictures have adopted with permission.

Conductive hydrogels enable closed-loop diagnostic–therapeutic systems by integrating real-time biosensing (e.g., biomarker detection via impedance/voltammetry) with adaptive therapeutic actions (e.g., on-demand drug release or electrostimulation) through programmable stimuli-responsiveness [119,120,121]. Their hybrid ionic–electronic networks continuously monitor physiological signals (e.g., glucose, pH) with high sensitivity, while stimuli-responsive components (e.g., pH-sensitive microgels, thermoresponsive polymers) trigger feedback-controlled therapy, such as drug release or electrical modulation. Dynamic covalent bonds (e.g., boronate esters) further allow in situ reconfiguration of mechanical/electrical properties to maintain tissue adhesion and signal fidelity during motion [122,123]. Ge et al. engineered an intelligent wound dressing (IWD) with integrated exudate management, sensing, and closed-loop therapy (Figure 9a). The system autonomously transports wound fluid via microfluidic channels while monitoring wound status through embedded sensors. Real-time data trigger smartphone-controlled LM heaters to activate thermoresponsive hydrogel-mediated drug release. In infected murine models, the IWD accelerated healing by modulating inflammatory pathways and stimulating regenerative processes, exemplifying a transformative closed-loop platform for adaptive wound care through synergistic bioelectronic integration [124]. Moreover, diabetic wounds are chronic lower limb lesions caused by hyperglycemia-induced vascular, neural, and immune dysfunction, with persistent inflammation, infections, and high amputation risk. Gong et al. engineered a wireless therapeutic–diagnostic patch integrating electroresponsive multifunctional hydrogels for closed-loop diabetic wound management (Figure 9b). This platform combines real-time glucose/pH monitoring with on-demand therapies (iontophoretic insulin delivery and electrical stimulation) through conductive, antimicrobial hydrogels that maintain an optimal healing microenvironment. Validated in animal models, the system demonstrated accelerated wound closure via coordinated inflammation modulation and tissue regeneration, establishing a paradigm for autonomous, personalized chronic wound care through adaptive bioelectronic-material synergy [125].

Conductive hydrogels are pioneering diagnostic–therapeutic integration in epilepsy management through adaptive bioelectronic interfaces that enable real-time seizure monitoring and closed-loop neuromodulation [126]. Qu et al. engineered a monolithic hydrogel-based neurotherapeutic platform featuring addressable microneedle electrode arrays for closed-loop epileptic seizure management (Figure 9c). This organic electronic system achieves spatiotemporally precise integration of electrophysiological recording and voltage-triggered drug release through conductive hydrogel-mediated biointerfacing. Upon detecting pathological neural activity, the device autonomously administers antiepileptic agents in a region-selective, dose-adaptive manner, achieving seizure suppression with drug dosages three orders of magnitude lower than conventional clinical approaches. The pharmaco-electrical synergy minimizes off-target effects while maintaining therapeutic efficacy, demonstrating a paradigm-shifting strategy for autonomous neurological disorder intervention through material-enabled bioelectronic feedback control [127].

## 4. Conclusions and Future Perspectives

The surging demand for wearable sensors in healthcare, human-machine interfaces, and personalized rehabilitation is driving unprecedented innovation in hydrogel-based flexible electronics. This review systematically summarizes structure–property relationships in advanced hydrogel composites and the emerging wearable applications of hydrogel sensors. The advancement of conductive hydrogels in wearable sensing hinges on strategic material innovations that harmonize conductivity, mechanical compliance, and multifunctionality. Biopolymer-based hydrogels (e.g., chitosan, CMC, gelatin) are emerging as a critical frontier, uniquely addressing biosafety and sustainability imperatives while enabling seamless integration with biological systems—features indispensable for implantable sensors and eco-friendly wearable technologies. Metal-based hydrogels (e.g., Ag/Au nanocomposites) prioritize high conductivity yet grapple with biocompatibility trade-offs, while carbon-based systems (graphene/CNTs) balance mechanical robustness with electrical performance through precise nanofiller dispersion. Conductive polymer hydrogels (e.g., PEDOT/PANI) excel in tunable redox activity for dynamic sensing, complemented by ionic hydrogels that mimic tissue softness via strain-insensitive ion transport, albeit challenged by environmental stability. Hybrid architectures transcend these limitations by synergizing multiple conductive phases (polymer–carbon–metal), unlocking unprecedented mechanical–electrical adaptability.

Despite remarkable progress, several challenges remain in terms of the long-term stability, reliable responsiveness under complex dynamic environments, biosafety, and scalable fabrication and integration of conductive hydrogels. Future research should focus on (1) developing hybrid conductive hydrogels: combining natural polymers (such as chitosan, cellulose, and alginate) with conductive nanomaterials (such as MXenes, carbon nanotubes, and graphene) to achieve a balanced combination of high conductivity, superior mechanical properties, and environmental durability. These hybrid systems show great promise for commercialization due to their tunable performance and biocompatibility, making them suitable for applications in wearable health monitoring and implantable devices. (2) Exploring bioinspired smart design strategies: drawing inspiration from biological systems to develop hydrogels capable of accurate perception and adaptive regulation of multidimensional stimuli. For instance, hydrogels mimicking the ion channels in nerve tissues or the hierarchical structures in natural skins can significantly improve the sensitivity and responsiveness of wearable sensors, enhancing their potential for commercialization in human–machine interfaces and soft robotics. (3) Promoting the deep integration of advanced manufacturing techniques: leveraging 3D/4D printing and microfluidic technologies to enable high-throughput, miniaturized, and scalable production of conductive hydrogel sensors. These techniques allow for precise customization of device architectures, paving the way for mass production and commercialization of flexible hydrogel-based electronics. (4) Enhancing multi-scale collaborative design: facilitating the co-design of materials, devices, and biological systems to accelerate the practical application of conductive hydrogels. Technologies such as self-healing and recyclable hydrogel systems can extend device lifespan and reduce maintenance costs, enhancing sustainability and supporting large-scale adoption in personalized health management, remote medical care, and smart rehabilitation.

## Figures and Tables

**Table 1:** Performance comparison of different types of conductive hydrogels.

Conductive Hydrogels	Mechanical Properties	Conductivity	Biocompatibility	Cost
Metal	High strength and flexibility due to metal reinforcement (e.g., nanoparticles or liquid metal).	Excellent (high electrical conductivity from metal nanoparticles or liquid metal).	Good (if using biocompatible metals like gold or silver; liquid metals may require coating).	High (due to the cost of metal nanoparticles or liquid metals).
Carbon	Moderate strength; brittle if not combined with polymers.	High (due to graphene, carbon nanotubes, or conductive polymers).	Moderate (carbon materials can cause inflammation if not properly functionalized).	Moderate to high (graphene and carbon nanotubes are expensive).
Conductive polymer	Moderate strength and flexibility; tunable based on polymer type and doping.	High (intrinsically conductive polymers like PEDOT:PSS or polypyrrole).	Good (can be tailored for biocompatibility; some polymers may require modification).	Moderate (conductive polymers are cheaper than metals or carbon materials).
Ionic	Soft and stretchable, but mechanical strength is often low.	Moderate (ionically conductive, but lower than electronic conductors).	Excellent (biocompatible and often used in biomedical applications).	Low (ionic hydrogels are typically made from inexpensive materials).
Hybrid	Combines strengths of components (e.g., high strength and flexibility).	High (combines ionic and electronic conductivity).	Good to excellent (depends on the combination of materials used).	Moderate to high (depends on the complexity and materials used).

## Data Availability

No new data were created or analyzed in this study. Data sharing is not applicable to this article.
